# Supplementary material for: Genome sequence and comparative analysis of reindeer (Rangifer tarandus) in northern Eurasia
Source: Sci Rep. 2020 Jun 2;10:8980. doi: 10.1038/s41598-020-65487-y (PMC7265531; doi:10.1038/s41598-020-65487-y)
Supplement: Supplementary file 1 — Supplementary information. [file 41598_2020_65487_MOESM1_ESM.docx]

Genome sequence and comparative analysis of reindeer (*Rangifer tarandus*) in Northern Eurasia.

Melak Weldenegodguad^1,2*^, Kisun Pokharel^1*^, Yao Ming^3*^, Mervi Honkatukia^1,4^, Jaana Peippo^1^, Tiina Reilas^1^, Knut Røed^5^ & Juha Kantanen^1**^

**Contents**

**Supplementary Data**

Supplementary Data 1. Reindeer-specific gene families. A total of 547 gene families were reindeer-specific compared to five ruminants. These reindeer-specific genes families comprised 1090 genes (sheet 1). Out of these 1090 genes, 802 had gene information (sheet 2).

Supplementary Data 2. Genes from reindeer-specific gene families that have known InterPro domains.

Supplementary Data 3. List of gene families expanded in reindeer. Altogether 2,683 genes were expanded in reindeer (sheet 1) of which 2,660 had gene information (sheet 2).

Supplementary Data 4. List of gene families contracted in reindeer.

Supplementary Data 5. Significantly enriched GO terms associated with expanded gene families.

Supplementary Data 6. Genes exhibiting signatures of positive selection.

**Supplementary Figures**

Figure S1. Estimation of genome size using 17-mer.

Figure S2. The GC content and sequencing depths of the reindeer genome.

Figure S3. GC content distribution for related species.

Figure S4. Comparison of the various gene parameters among sequenced mammalian genomes (*R.tarandus*, *H. sapiens, M. musculus, B. taurus, C. familiaris and C. dromedaries* ).

**Supplementary Tables**

Table S1. Statistics of raw and clean data.

Table S2. K- mer statistics.

Table S3. Statistics of the assembled sequence length.

Table S4. Assessment of the completeness of coding region using *de novo* transcriptome assembly of reindeer.

Table S5. Summary of BUSCO for genome assembly.

Table S6. Summary of BUSCO for gene set.

Table S7. General statistics of predicted protein-coding genes.

Table S8. Statistics of functional annotations. Four protein databases (InterPro, KEGG, Swissprot and TrEMBL) were used for predicting gene functions. The table shows numbers of genes match to each database. The TrEMBL results were based on five homologous species and exclude unknown or hypothetical genes.

Table S9. Summary of non-coding RNA genes in the genome.

Table S10.General statistics of repeats in genome.

Table S11. Contents of transposable elements (TEs) in reindeer genome.

Table S12. Summary of gene families of reindeer and nine other mammalian genomes.

Table S13. List of significantly enriched GO terms associated with reindeer-specific gene families. Two types of GO categories, molecular function (MF) and biological processes (BP) have been considered in this study. In the table “Query item” refers to number of reindeer-specific genes.

Table S14. Top 30 significantly enriched GO terms associated with expanded gene families. In the table “Query item” refers to number of expanded genes.

Table S15. List of significantly enriched GO terms associated with genes under positive selection in reindeer. In the table “Query item” refers to number of positively selected genes.

Table S16. Summary of clean reads generated from whole-genome resequencing of 23 reindeer samples.

Table S17. Summary statistics of the mapped reads for each resequencing sample.

Table S18. Summary statistics of the variants identified in each resequencing sample.

Table S19. Annotation of SNPs.

Table S20. Mitochondrial genome annotation.

**Supplementary figures**


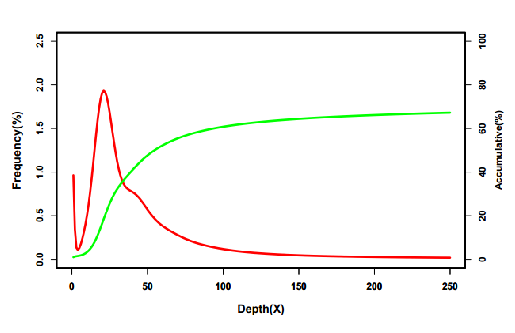


**Figure S1. Estimation of genome size using 17-mer.**


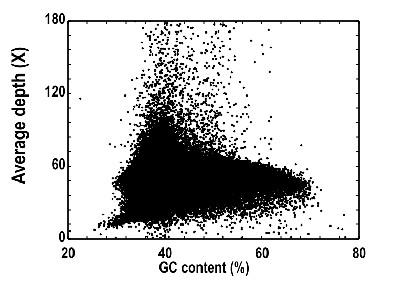


**Figure S2. The GC content and sequencing depths of the reindeer genome.**


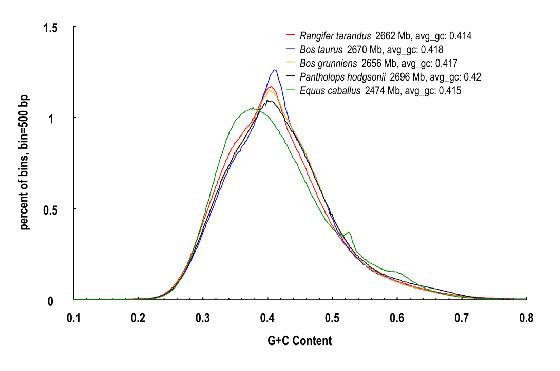


**Figure S3. GC content distribution for related species.**

**
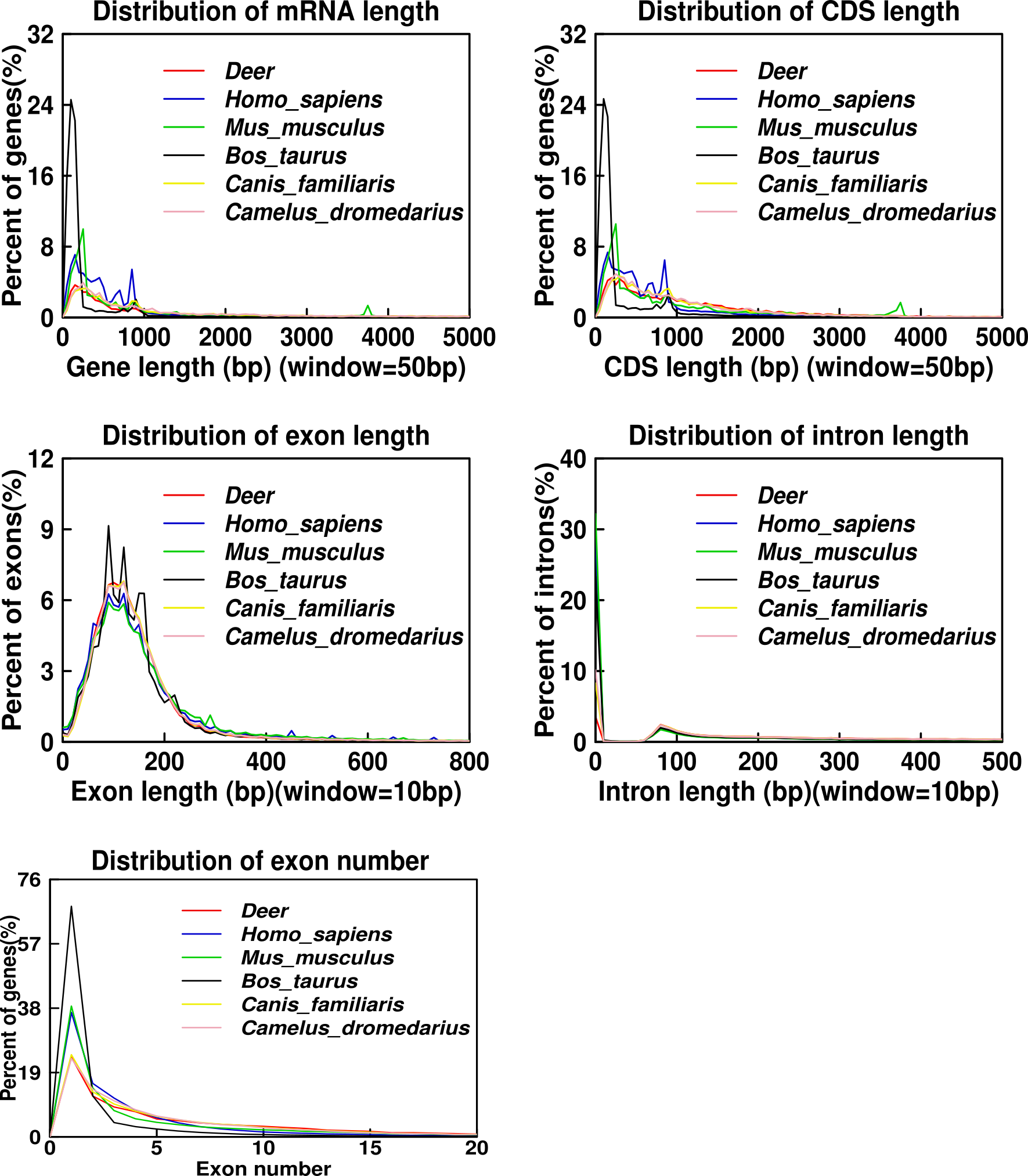
**

**Figure S4 Comparison of the various gene parameters among sequenced mammalian genomes (*R. tarandus*, *H. sapiens, M. musculus, B. taurus, C. familiaris and C. dromedaries*).**

**Supplementary tables**

**Table S1: Statistics of raw and clean data.**

|  |  |  | **Raw data** | | | **Clean data^1^** | | |
| --- | --- | --- | --- | --- | --- | --- | --- | --- |
|  | Insert size | Read Length (bp) | Data (Mb) | Sequence depth (X) |  | Data (Mb) | Sequence depth (X) |  |
|  | 170 | 125 | 68,697 | 22.9 |  | 66,229 | 22 |  |
|  | 500 | 125 | 66,443 | 22.1 |  | 64,721 | 21.6 |  |
|  | 800 | 125 | 52,072 | 17.3 |  | 50,437 | 16.8 |  |
|  | 2000 | 125 | 71,203 | 23.7 |  | 35,795 | 11.9 |  |
|  | 5000 | 125 | 69,208 | 23.1 |  | 30,798 | 10.2 |  |
|  | 10000 | 50 | 121,472 | 40.5 |  | 34,394 | 11.5 |  |
|  | 20000 | 50 | 63,897 | 21.2 |  | 18,173 | 6 |  |
| Total |  |  | 512,992 | 170 |  | 300,547 | 100 |  |

^1^ Clean data were generated by filtering low-quality reads.

**Table S2: K- mer statistics.**

| **K** | **K-mer_num** | **Peak_depth** | **Genome Size** | **Used Bases** | **Used Reads** | **X** |
| --- | --- | --- | --- | --- | --- | --- |
| 17 | 55,451,706,382 | 19 | 2,918,510,862 | 64,695,044,125 | 517,560,353 | 21.6 |

**Table S3: Statistics of the assembled sequence length.**

|  | **Contig** | | **Scaffold** | |
| --- | --- | --- | --- | --- |
|  | **Length (bp)** | **Number** | **Length (bp)** | **Number** |
| maximum length | 444,581 |  | 28,190,176 |  |
| N10 | 127,487 | 1,583 | 13,328,327 | 17 |
| N20 | 95,147 | 3,989 | 9,453,896 | 41 |
| N30 | 75,378 | 7,096 | 7,772,072 | 72 |
| N40 | 60,833 | 10,976 | 6,547,969 | 110 |
| N50 | 48,758 | 15,793 | 5,023,617 | 157 |
| N60 | 38,674 | 21,821 | 3,905,468 | 217 |
| N70 | 29,450 | 29,560 | 2,815,736 | 297 |
| N80 | 20,553 | 40,140 | 1,868,990 | 412 |
| N90 | 10,934 | 57,163 | 839,408 | 624 |
| Total length | 2,619,059,166 |  | 2,662,986,674 |  |
| number >=100bp |  | 256,454 |  | 131,360 |
| number >=2000bp |  | 89,211 |  | 4,970 |

**Table S4: Assessment of the completeness of coding region using *de novo* transcriptome assembly of reindeer.**

| **Length of transcript** | **Number** | **Total length (bp)** | **Covered by assembly** | **With > 90% sequence in one scaffold** | | **With > 50% sequence in one scaffold** | |
| --- | --- | --- | --- | --- | --- | --- | --- |
|  |  |  |  | **Number** | **Percent (%)** | **Number** | **Percent (%)** |
| **>0bp** | 94,673 | 91,938,826 | 99.80 | 92.392 | 97.59 | 94,347 | 99.65 |
| **>200bp** | 94,673 | 91,938,826 | 99.80 | 92.392 | 97.59 | 94,347 | 99.65 |
| **>500bp** | 42,796 | 76,471,914 | 99.87 | 41,400 | 96.73 | 42,695 | 99.67 |
| **>1000bp** | 26,599 | 65,090,161 | 99.92 | 25,587 | 96.19 | 26,531 | 99.74 |

**Table S5: Summary of BUSCO for genome assembly.**

|  | **Number** | **Percent (%)** |
| --- | --- | --- |
| **Complete BUSCOs** | 3895 | 94.9 |
| **Complete and single-copy BUSCOs** | 3814 | 92.9 |
| **Complete and duplicated BUSCOs** | 216 | 7.14 |
| **Fragmented BUSCOs** | 81 | 2.0 |
| **Missing BUSCOs** | 100 | 2.7 |
| **Total BUSCO groups searched** | 4104 |  |

**Table S6: Summary of BUSCO for gene set.**

|  | **Number** | **Percent (%)** |
| --- | --- | --- |
| **Complete BUSCOs** | 3973 | 96.8 |
| **Complete and single-copy BUSCOs** | 3861 | 94.1 |
| **Complete and duplicated BUSCOs** | 112 | 2.7 |
| **Fragmented BUSCOs** | 36 | 0.9 |
| **Missing BUSCOs** | 95 | 2.3 |
| **Total BUSCO groups searched** | 4104 |  |

**Table S7: General statistics of predicted protein-coding genes.**

| **Gene set** |  | **Number** | **Average transcript length (bp)** | **Average CDS length (bp)** | **Average exon per gene** | **Average exon length (bp)** | **Average intron length (bp)** |
| --- | --- | --- | --- | --- | --- | --- | --- |
| **Homolog** | *H.sapiens* | 26,607 | 20,492 | 1,290 | 7.2 | 177 | 3,068 |
|  | *M.musculus* | 24,330 | 19,855 | 1,314 | 7.3 | 177 | 2,902 |
|  | *B.taurus* | 27,782 | 19,574 | 1,270 | 7.3 | 173 | 2,903 |
|  | *C.dromedarius* | 28,615 | 20,007 | 1,248 | 7.2 | 173 | 3,018 |
|  | *C.familiaris* | 26,234 | 18,653 | 1,219 | 6.9 | 175 | 2,920 |
| **RNA** |  | 45,329 | 17,880 | 5,603 | 12 | 448 | 1,211 |
| **Final set** |  | 27,332 | 28,808 | 1,305 | 7.4 | 175 | 3,804 |

**Table S8: Statistics of functional annotations.** Four protein databases (InterPro, KEGG, Swissprot and TrEMBL) were used for predicting gene functions. The table shows numbers of genes match to each database. The TrEMBL results were based on five homologous species and exclude unknown or hypothetical genes.

|  | **Number** | **Percent (%)** |
| --- | --- | --- |
| **Total** | 27,332 |  |
| **InterPro** | 25,241 | 92.35 |
| **KEGG** | 22,564 | 82.56 |
| **Swissprot** | 25,928 | 94.86 |
| **TrEMBL** | 26,707 | 97.71 |
| **Annotated** | 26,838 | 98.19 |
| **Unannotated** | 494 | 1.81 |

**Table S9: Summary of non-coding RNA genes in the genome.**

| **Type** |  | **Copy number** | **Average length(bp)** | **Total length (bp)** | **% of genome** |
| --- | --- | --- | --- | --- | --- |
| **miRNA** |  | 551 | 89.81 | 49,487 | 0.001858 |
| **tRNA** |  | 3,994 | 72.97 | 725,391 | 0.02724 |
| **rRNA** | **rRNA** | 329 | 109.08 | 35,887 | 0.001348 |
|  | **18S** | 97 | 85.72 | 8,315 | 0.000312 |
|  | **28S** | 223 | 120.94 | 26,970 | 0.001013 |
|  | **5.8S** | 6 | 66.00 | 396 | 0.000015 |
|  | **5S** | 3 | 68.67 | 206 | 0.000008 |
| **snRNA** | **snRNA** | 1,706 | 111.51 | 190,237 | 0.007144 |
|  | **CD-box** | 359 | 93.57 | 33,592 | 0.001261 |
|  | **HACA-box** | 310 | 134.61 | 41,729 | 0.001567 |
|  | **splicing** | 1,009 | 110.58 | 111,573 | 0.00419 |

**Table S10: General statistics of repeats in genome.**

| **Type** | **Repeat length (bp)** | **% of genome length** |
| --- | --- | --- |
| **Trf** | 53,294,515 | 2.00 |
| **Repeatmasker** | 857,864,301 | 32.21 |
| **Proteinmask** | 290,364,346 | 10.90 |
| ***De novo*** | 874,929,123 | 32.85 |
| **Total** | 1,012,556,108 | 38.02 |

**Table S11: Contents of transposable elements (TEs) in reindeer genome.**

|  | **Repbase TEs** | | **TE proteins** | | ***De novo*** | | **Combined TEs** | |
| --- | --- | --- | --- | --- | --- | --- | --- | --- |
| **Type** | **length (bp)** | **% genome** | **length (bp)** | **% genome** | **length (bp)** | **% genome** | **length (bp)** | **% genome** |
| **DNA** | 31,664,761 | 1.18 | 4,619,577 | 0.17 | 34,692,623 | 1.30 | 40,055,380 | 1.84 |
| **LINE** | 622,392,940 | 23.37 | 276,784,311 | 10.39 | 675,338,320 | 25.36 | 745,462,986 | 27.99 |
| **SINE** | 132,576,480 | 4.97 | 0 | 0 | 43,027,594 | 1.61 | 149,586,647 | 5.61 |
| **LTR** | 83,394,380 | 3.13 | 9,081,381 | 0.34 | 87,938,964 | 3.30 | 115,391,495 | 4.33 |
| **Other** | 928 | 0.000035 | 0 | 0 | 0 | 0 | 928 | 0.000035 |
| **Unknown** | 0 | 0 | 0 | 0 | 45,329,565 | 1.70 | 45,329,565 | 1.70 |
| **Total** | 857,864,301 | 32.21 | 290,364,346 | 10.90 | 870,320,451 | 32.68 | 979,488,943 | 36.78 |

Note: Repbase TEs: the result of RepeatMasker based on Repbase; TE proteins: the result of RepeatProteinMask based on Repbase; *De novo*: Result of RepeatMasker by using library predicted through *De novo*; Combined: combine the results of Repbase TEs, TE proteins and *De novo*.

**Table S12: Summary of gene families of reindeer and nine other mammalian genomes.**

| Species | Total genes | Unclustered genes | Families | Unique families | Ave. genes per family |
| --- | --- | --- | --- | --- | --- |
| *R. tarandus* | 27332 | 2224 | 17709 | 226 | 1.47 |
| *C. dromedaries* | 19292 | 2084 | 15751 | 51 | 1.09 |
| *C. hircus* | 22175 | 2378 | 16887 | 22 | 1.17 |
| *O. aries* | 20889 | 548 | 17270 | 29 | 1.18 |
| *B. taurus* | 19970 | 114 | 17040 | 1 | 1.17 |
| *B. grunniens* | 22282 | 578 | 17522 | 25 | 1.24 |
| *E. caballus* | 20419 | 278 | 16617 | 21 | 1.21 |
| *C. familiaris* | 19839 | 785 | 16835 | 15 | 1.13 |
| *U. maritimus* | 21142 | 758 | 16769 | 60 | 1.22 |
| *H. sapiens* | 20071 | 627 | 18088 | 112 | 1.07 |

**Table S13: List of significantly enriched GO terms associated with reindeer-specific gene families**. Two types of GO categories, molecular function (MF) and biological processes (BP) have been considered in this study. In the table “Query item” refers to number of reindeer-specific genes.

| **GO ID** | **Term type** | **Term** | **Query item** | **P-value** | **FDR** |
| --- | --- | --- | --- | --- | --- |
| GO:0050136 | MF | NADH dehydrogenase (quinone) activity | 7 | 9.2E-07 | 0.00011 |
| GO:0008137 | MF | NADH dehydrogenase (ubiquinone) activity | 7 | 9.2E-07 | 0.00011 |
| GO:0016655 | MF | oxidoreductase activity, acting on NAD(P)H, quinone or similar compound as acceptor | 7 | 9.2E-07 | 0.00011 |
| GO:0099600 | MF | transmembrane receptor activity | 47 | 3.3E-06 | 0.00038 |
| GO:0010556 | BP | regulation of macromolecule biosynthetic process | 52 | 3E-06 | 0.00049 |
| GO:0003954 | MF | NADH dehydrogenase activity | 7 | 4.5E-06 | 0.00052 |
| GO:0031326 | BP | regulation of cellular biosynthetic process | 52 | 3.5E-06 | 0.00058 |
| GO:0009889 | BP | regulation of biosynthetic process | 52 | 3.6E-06 | 0.00059 |
| GO:2000112 | BP | regulation of cellular macromolecule biosynthetic process | 51 | 5.2E-06 | 0.00085 |
| GO:0097659 | BP | nucleic acid-templated transcription | 55 | 6E-06 | 0.00097 |
| GO:0006351 | BP | transcription, DNA-templated | 55 | 6E-06 | 0.00097 |
| GO:0010468 | BP | regulation of gene expression | 51 | 6.2E-06 | 0.001 |
| GO:1903506 | BP | regulation of nucleic acid-templated transcription | 50 | 6.6E-06 | 0.0011 |
| GO:0006355 | BP | regulation of transcription, DNA-templated | 50 | 6.6E-06 | 0.0011 |
| GO:2001141 | BP | regulation of RNA biosynthetic process | 50 | 6.7E-06 | 0.0011 |
| GO:0051252 | BP | regulation of RNA metabolic process | 50 | 7.1E-06 | 0.0012 |
| GO:0032774 | BP | RNA biosynthetic process | 55 | 7.1E-06 | 0.0012 |
| GO:0051171 | BP | regulation of nitrogen compound metabolic process | 51 | 7.3E-06 | 0.0012 |
| GO:0019219 | BP | regulation of nucleobase-containing compound metabolic process | 50 | 0.00001 | 0.0017 |
| GO:0004888 | MF | transmembrane signaling receptor activity | 43 | 1.8E-05 | 0.0021 |
| GO:0080090 | BP | regulation of primary metabolic process | 52 | 1.4E-05 | 0.0022 |
| GO:0060255 | BP | regulation of macromolecule metabolic process | 52 | 1.5E-05 | 0.0024 |
| GO:0031323 | BP | regulation of cellular metabolic process | 52 | 1.6E-05 | 0.0026 |
| GO:0017137 | MF | Rab GTPase binding | 5 | 2.7E-05 | 0.0031 |
| GO:0019222 | BP | regulation of metabolic process | 52 | 2.2E-05 | 0.0035 |
| GO:0004871 | MF | signal transducer activity | 47 | 0.00004 | 0.0046 |
| GO:0004984 | MF | olfactory receptor activity | 16 | 5.9E-05 | 0.0068 |
| GO:0034654 | BP | nucleobase-containing compound biosynthetic process | 57 | 4.4E-05 | 0.0072 |
| GO:0060089 | MF | molecular transducer activity | 47 | 6.3E-05 | 0.0073 |
| GO:0004872 | MF | receptor activity | 47 | 6.3E-05 | 0.0073 |
| GO:0038023 | MF | signaling receptor activity | 43 | 6.5E-05 | 0.0075 |
| GO:0019438 | BP | aromatic compound biosynthetic process | 57 | 6.8E-05 | 0.011 |
| GO:0018130 | BP | heterocycle biosynthetic process | 57 | 7.4E-05 | 0.012 |
| GO:1901362 | BP | organic cyclic compound biosynthetic process | 57 | 8.7E-05 | 0.014 |
| GO:0004867 | MF | serine-type endopeptidase inhibitor activity | 8 | 0.00012 | 0.014 |
| GO:0004950 | MF | chemokine receptor activity | 5 | 0.00029 | 0.034 |
| GO:0001637 | MF | G-protein coupled chemoattractant receptor activity | 5 | 0.00029 | 0.034 |
| GO:0007186 | BP | G-protein coupled receptor signaling pathway | 35 | 0.00028 | 0.046 |
| GO:0046872 | MF | metal ion binding | 89 | 0.0004 | 0.047 |

MF: molecular function; BP: biological process

**Table S14: Top 30 significantly enriched GO terms associated with expanded gene families.** In the table “Query item” refers to number of expanded genes.

| **GO ID** | **Term type** | **Term** | **Query item** | **P-value** | **FDR** |
| --- | --- | --- | --- | --- | --- |
| GO:0003735 | MF | structural constituent of ribosome | 651 | 0 | 0 |
| GO:0006412 | BP | translation | 683 | 1.5e-308 | 6.8E-306 |
| GO:0043043 | BP | peptide biosynthetic process | 683 | 2.4E-306 | 1.1E-303 |
| GO:0043604 | BP | amide biosynthetic process | 683 | 1.9E-305 | 8.7E-303 |
| GO:0006518 | BP | peptide metabolic process | 683 | 1.1E-297 | 4.9E-295 |
| GO:0043603 | BP | cellular amide metabolic process | 683 | 1E-292 | 4.9E-290 |
| GO:1901566 | BP | organonitrogen compound biosynthetic process | 726 | 8.8E-268 | 4.1E-265 |
| GO:0005198 | MF | structural molecule activity | 654 | 2.6E-258 | 5.3E-256 |
| GO:1901564 | BP | organonitrogen compound metabolic process | 751 | 2.5E-225 | 1.2E-222 |
| GO:0044271 | BP | cellular nitrogen compound biosynthetic process | 908 | 4.6E-139 | 2.2E-136 |
| GO:0034645 | BP | cellular macromolecule biosynthetic process | 869 | 2.8E-126 | 1.3E-123 |
| GO:0009059 | BP | macromolecule biosynthetic process | 869 | 2.3E-124 | 1.1E-121 |
| GO:0010467 | BP | gene expression | 892 | 2.4E-124 | 1.1E-121 |
| GO:0044249 | BP | cellular biosynthetic process | 920 | 1.2E-114 | 5.5E-112 |
| GO:1901576 | BP | organic substance biosynthetic process | 915 | 4.5E-111 | 2.1E-108 |
| GO:0044267 | BP | cellular protein metabolic process | 809 | 5.9E-108 | 2.8E-105 |
| GO:0009058 | BP | biosynthetic process | 920 | 7E-107 | 3.3E-104 |
| GO:0034641 | BP | cellular nitrogen compound metabolic process | 967 | 7.6E-98 | 3.6E-95 |
| GO:0006807 | BP | nitrogen compound metabolic process | 967 | 4.8E-87 | 2.2E-84 |
| GO:0019538 | BP | protein metabolic process | 822 | 1.9E-73 | 9.1E-71 |
| GO:0008199 | MF | ferric iron binding | 84 | 2E-59 | 4.2E-57 |
| GO:0006826 | BP | iron ion transport | 84 | 7E-56 | 3.3E-53 |
| GO:0006879 | BP | cellular iron ion homeostasis | 84 | 1.5E-53 | 6.9E-51 |
| GO:0046916 | BP | cellular transition metal ion homeostasis | 84 | 1.5E-53 | 6.9E-51 |
| GO:0055072 | BP | iron ion homeostasis | 84 | 1.5E-53 | 6.9E-51 |
| GO:0044260 | BP | cellular macromolecule metabolic process | 1024 | 3.4E-53 | 1.6E-50 |
| GO:0055076 | BP | transition metal ion homeostasis | 84 | 7.9E-53 | 3.7E-50 |
| GO:0000041 | BP | transition metal ion transport | 84 | 1.9E-51 | 9E-49 |
| GO:0006875 | BP | cellular metal ion homeostasis | 84 | 1.4E-46 | 6.4E-44 |
| GO:0006873 | BP | cellular ion homeostasis | 84 | 4.8E-46 | 2.2E-43 |

MF: molecular function; BP: biological process

**Table S15: List of significantly enriched GO terms associated with genes under positive selection in reindeer.** In the table “Query item” refers to number of positively selected genes.

| **GO ID** | **Term type** | **Term** | **Query item** | **P-value** | **FDR** |
| --- | --- | --- | --- | --- | --- |
| GO:0005272 | MF | sodium channel activity | 5 | 0.00013 | 0.013 |
| GO:0022803 | MF | passive transmembrane transporter activity | 20 | 0.00015 | 0.016 |
| GO:0015267 | MF | channel activity | 20 | 0.00015 | 0.016 |
| GO:0022835 | MF | transmitter-gated channel activity | 5 | 0.0002 | 0.021 |
| GO:0022824 | MF | transmitter-gated ion channel activity | 5 | 0.0002 | 0.021 |
| GO:0005216 | MF | ion channel activity | 19 | 0.0004 | 0.041 |
| GO:0022838 | MF | substrate-specific channel activity | 19 | 0.0004 | 0.041 |
| GO:0030594 | MF | neurotransmitter receptor activity | 5 | 0.0011 | 0.11 |
| GO:0005085 | MF | guanyl-nucleotide exchange factor activity | 10 | 0.0017 | 0.17 |
| GO:0006820 | BP | anion transport | 9 | 0.0017 | 0.24 |
| GO:0006814 | BP | sodium ion transport | 6 | 0.0017 | 0.25 |
| GO:0035556 | BP | intracellular signal transduction | 29 | 0.0019 | 0.28 |
| GO:0005230 | MF | extracellular ligand-gated ion channel activity | 7 | 0.0029 | 0.3 |
| GO:0022836 | MF | gated channel activity | 14 | 0.0032 | 0.33 |
| GO:0008509 | MF | anion transmembrane transporter activity | 7 | 0.0035 | 0.37 |
| GO:0035091 | MF | phosphatidylinositol binding | 6 | 0.0038 | 0.39 |
| GO:0008270 | MF | zinc ion binding | 38 | 0.0041 | 0.42 |
| GO:0005543 | MF | phospholipid binding | 7 | 0.0055 | 0.57 |
| GO:0008236 | MF | serine-type peptidase activity | 10 | 0.0057 | 0.59 |
| GO:0017171 | MF | serine hydrolase activity | 10 | 0.0057 | 0.59 |
| GO:0004252 | MF | serine-type endopeptidase activity | 9 | 0.0069 | 0.72 |
| GO:0007166 | BP | cell surface receptor signaling pathway | 15 | 0.0099 | 1 |
| GO:0015103 | MF | inorganic anion transmembrane transporter activity | 5 | 0.01 | 1 |
| GO:0022892 | MF | substrate-specific transporter activity | 30 | 0.011 | 1 |
| GO:0005215 | MF | transporter activity | 37 | 0.011 | 1 |
| GO:0007265 | BP | Ras protein signal transduction | 7 | 0.011 | 1 |
| GO:0051716 | BP | cellular response to stimulus | 70 | 0.012 | 1 |
| GO:0015698 | BP | inorganic anion transport | 5 | 0.012 | 1 |
| GO:0007264 | BP | small GTPase mediated signal transduction | 18 | 0.012 | 1 |
| GO:0015276 | MF | ligand-gated ion channel activity | 7 | 0.013 | 1 |
| GO:0005515 | MF | protein binding | 146 | 0.013 | 1 |
| GO:0022834 | MF | ligand-gated channel activity | 7 | 0.013 | 1 |
| GO:0007154 | BP | cell communication | 67 | 0.013 | 1 |
| GO:0044700 | BP | single organism signaling | 66 | 0.014 | 1 |
| GO:0007267 | BP | cell-cell signaling | 8 | 0.014 | 1 |
| GO:0023052 | BP | signaling | 66 | 0.015 | 1 |
| GO:0007165 | BP | signal transduction | 64 | 0.017 | 1 |
| GO:0015075 | MF | ion transmembrane transporter activity | 25 | 0.023 | 1 |
| GO:0022891 | MF | substrate-specific transmembrane transporter activity | 27 | 0.023 | 1 |
| GO:0016773 | MF | phosphotransferase activity, alcohol group as acceptor | 27 | 0.024 | 1 |
| GO:1902589 | BP | single-organism organelle organization | 13 | 0.025 | 1 |
| GO:0016301 | MF | kinase activity | 27 | 0.031 | 1 |
| GO:0070011 | MF | peptidase activity, acting on L-amino acid peptides | 21 | 0.031 | 1 |
| GO:0043167 | MF | ion binding | 80 | 0.035 | 1 |
| GO:0006811 | BP | ion transport | 26 | 0.036 | 1 |
| GO:0005261 | MF | cation channel activity | 10 | 0.037 | 1 |
| GO:0051056 | BP | regulation of small GTPase mediated signal transduction | 6 | 0.038 | 1 |
| GO:0008233 | MF | peptidase activity | 21 | 0.04 | 1 |
| GO:0006810 | BP | transport | 58 | 0.04 | 1 |
| GO:0015081 | MF | sodium ion transmembrane transporter activity | 5 | 0.041 | 1 |
| GO:0051234 | BP | establishment of localization | 58 | 0.041 | 1 |
| GO:0008610 | BP | lipid biosynthetic process | 5 | 0.041 | 1 |
| GO:0004518 | MF | nuclease activity | 5 | 0.044 | 1 |
| GO:0046872 | MF | metal ion binding | 77 | 0.045 | 1 |
| GO:0050896 | BP | response to stimulus | 76 | 0.045 | 1 |
| GO:0043169 | MF | cation binding | 77 | 0.049 | 1 |

MF: molecular function; BP: biological process

**Table S16: Summary of clean reads generated from whole-genome resequencing of 23 reindeer samples.**

| **Sample name** | **Type** | **Country** | **Code** | **Read length (bp)** | **Clean reads** | **Clean bases** | **Q20(%)** | **GC(%)** |
| --- | --- | --- | --- | --- | --- | --- | --- | --- |
| NMBU-23 | Russian domestic forest reindeer | Russia | Ru-D-F | 150 | 197129978 | 29569496700 | 97.01;92.13 | 41,93 |
| NMBU-24 | Russian domestic forest reindeer | Russia | Ru-D-F | 150 | 197224378 | 29583656700 | 97.01;92.23 | 42,44 |
| NMBU-25 | Fennoscandian domestic tundra reindeer | Norway | Fe-D-T | 150 | 196946404 | 29541960600 | 96.73;92.18 | 43,25 |
| NMBU-26 | Fennoscandian domestic tundra reindeer | Norway | Fe-D-T | 150 | 197200970 | 29580145500 | 96.83;92.14 | 43,03 |
| NMBU-27 | Russian wild arctic reindeer (i.e. Novaya Zemlya) | Russia | Ru-W-A | 150 | 197170264 | 29575539600 | 97.20;91.94 | 43,83 |
| NMBU-28 | Russian wild arctic reindeer (i.e. Novaya Zemlya) | Russia | Ru-W-A | 150 | 197641802 | 29646270300 | 97.71;90.76 | 43,4 |
| NMBU-29 | Russian wild tundra reindeer | Russia | Ru-W-T | 150 | 197511096 | 29626664400 | 97.72;91.91 | 43,95 |
| NMBU-30 | Russian wild tundra reindeer | Russia | Ru-W-T | 150 | 197275212 | 29591281800 | 97.78;92.42 | 43,6 |
| NMBU-31 | Russian wild tundra reindeer | Russia | Ru-W-T | 150 | 197478826 | 29621823900 | 97.79;91.98 | 43,66 |
| NMBU-32 | Russian wild tundra reindeer | Russia | Ru-W-T | 150 | 197208290 | 29581243500 | 92.34;94.31 | 43,88 |
| NMBU-33 | Fennoscandian domestic tundra reindeer | Norway | Fe-D-T | 150 | 195739934 | 29360990100 | 91.59;93.76 | 44,52 |
| NMBU-34 | Fennoscandian domestic tundra reindeer | Norway | Fe-D-T | 150 | 196758758 | 29513813700 | 92.18;94.15 | 44,57 |
| NMBU-35 | Svalbard wild arctic reindeer | Svalbard (Norway) | Sv-W-A | 150 | 195509386 | 29326407900 | 91.82;93.89 | 44,42 |
| NMBU-36 | Svalbard wild arctic reindeer | Svalbard (Norway) | Sv-W-A | 150 | 196518502 | 29477775300 | 97.74;92.78 | 43,86 |
| NMBU-37 | Svalbard wild arctic reindeer | Svalbard (Norway) | Sv-W-A | 150 | 196560146 | 29484021900 | 97.67;92.57 | 43,9 |
| NMBU-38 | Fennoscandian wild tundra reindeer | Norway | Fe-W-T | 150 | 196509916 | 29476487400 | 97.62;92.73 | 44,42 |
| NMBU-39 | Fennoscandian wild tundra reindeer | Norway | Fe-W-T | 150 | 196012330 | 29401849500 | 97.66;92.76 | 44,07 |
| NMBU-40 | Fennoscandian wild tundra reindeer | Norway | Fe-W-T | 150 | 196752758 | 29512913700 | 96.96;93.57 | 44,23 |
| NMBU-41 | Fennoscandian wild tundra reindeer | Norway | Fe-W-T | 150 | 198418656 | 29762798400 | 96.98;93.59 | 43,87 |
| NMBU-42 | Fennoscandian wild tundra reindeer | Norway | Fe-W-T | 150 | 198495940 | 29774391000 | 96.88;92.90 | 43,8 |
| NMBU-43 | Alaska domestic tundra  reindeer | USA (Alaska) | Al-D-T | 150 | 196678634 | 29501795100 | 96.95;93.81 | 45,26 |
| NMBU-44 | Alaska domestic tundra  reindeer | USA (Alaska) | Al-D-T | 150 | 196889532 | 29533429800 | 97.18;91.70 | 45,2 |
| NMBU-45 | Alaska wild caribou | USA (Alaska) | Al-W-C | 150 | 196444314 | 29466647100 | 97.15;91.83 | 44,47 |

**Table S17: Summary statistics of the mapped reads for each resequencing sample.**

| **Sample name** | **Type** | **Country** | **Code** | **Mapped (%)** |
| --- | --- | --- | --- | --- |
| NMBU-23 | Russian domestic forest reindeer | Russia | Ru-D-F | 98,79 |
| NMBU-24 | Russian domestic forest reindeer | Russia | Ru-D-F | 98,56 |
| NMBU-25 | Fennoscandian domestic tundra reindeer | Norway | Fe-D-T | 99,18 |
| NMBU-26 | Fennoscandian domestic tundra reindeer | Norway | Fe-D-T | 99,09 |
| NMBU-27 | Russian wild arctic reindeer (i.e. Novaya Zemlya) | Russia | Ru-W-A | 99,28 |
| NMBU-28 | Russian wild arctic reindeer (i.e. Novaya Zemlya) | Russia | Ru-W-A | 98,21 |
| NMBU-29 | Russian wild tundra reindeer | Russia | Ru-W-T | 98,42 |
| NMBU-30 | Russian wild tundra reindeer | Russia | Ru-W-T | 99,08 |
| NMBU-31 | Russian wild tundra reindeer | Russia | Ru-W-T | 99 |
| NMBU-32 | Russian wild tundra reindeer | Russia | Ru-W-T | 98,54 |
| NMBU-33 | Fennoscandian domestic tundra reindeer | Norway | Fe-D-T | 99,09 |
| NMBU-34 | Fennoscandian domestic tundra reindeer | Norway | Fe-D-T | 99,09 |
| NMBU-35 | Svalbard wild arctic reindeer | Svalbard (Norway) | Sv-W-A | 98,87 |
| NMBU-36 | Svalbard wild arctic reindeer | Svalbard (Norway) | Sv-W-A | 98,84 |
| NMBU-37 | Svalbard wild arctic reindeer | Svalbard (Norway) | Sv-W-A | 98,83 |
| NMBU-38 | Fennoscandian wild tundra reindeer | Norway | Fe-W-T | 98,1 |
| NMBU-39 | Fennoscandian wild tundra reindeer | Norway | Fe-W-T | 99,12 |
| NMBU-40 | Fennoscandian wild tundra reindeer | Norway | Fe-W-T | 98,98 |
| NMBU-41 | Fennoscandian wild tundra reindeer | Norway | Fe-W-T | 98,56 |
| NMBU-42 | Fennoscandian wild tundra reindeer | Norway | Fe-W-T | 98,86 |
| NMBU-43 | Alaska domestic tundra reindeer | USA (Alaska) | Al-D-T | 98,58 |
| NMBU-44 | Alaska domestic tundra reindeer | USA (Alaska) | Al-D-T | 97,97 |
| NMBU-45 | Alaska wild caribou | USA (Alaska) | Al-W-C | 98,99 |

**Table S18: Summary statistics of the variants identified in each resequencing sample.**

| **Sample name** | **Code** | **Number of SNPs** | **Number of heterozygous SNPs** | **Number of homozygous SNPs** | **Number of indels** |
| --- | --- | --- | --- | --- | --- |
| NMBU-23 | Ru-D-F | 8029264 | 4676488 | 3352776 | 1014986 |
| NMBU-24 | Ru-D-F | 7977352 | 4455208 | 3522144 | 997145 |
| NMBU-25 | Fe-D-T | 6902853 | 3811778 | 3091075 | 843556 |
| NMBU-26 | Fe-D-T | 7017232 | 3864203 | 3153029 | 868310 |
| NMBU-27 | Ru-W-A | 7408460 | 3459260 | 3949200 | 923800 |
| NMBU-28 | Ru-W-A | 7499726 | 3629870 | 3869856 | 927347 |
| NMBU-29 | Ru-W-T | 7854635 | 4434727 | 3419908 | 955428 |
| NMBU-30 | Ru-W-T | 7679475 | 4114040 | 3565435 | 938555 |
| NMBU-31 | Ru-W-T | 7741624 | 4222697 | 3518927 | 945422 |
| NMBU-32 | Ru-W-T | 7434899 | 3786060 | 3648839 | 933342 |
| NMBU-33 | Fe-D-T | 6656663 | 3552086 | 3104577 | 826515 |
| NMBU-34 | Fe-D-T | 6580652 | 3504009 | 3076643 | 821481 |
| NMBU-35 | Sv-W-A | 6641877 | 1372091 | 5269786 | 866670 |
| NMBU-36 | Sv-W-A | 6736833 | 1327658 | 5409175 | 842797 |
| NMBU-37 | Sv-W-A | 6786081 | 1417957 | 5368124 | 848290 |
| NMBU-38 | Fe-W-T | 6943964 | 3777025 | 3166939 | 827401 |
| NMBU-39 | Fe-W-T | 7063533 | 3985083 | 3078450 | 841937 |
| NMBU-40 | Fe-W-T | 7103364 | 3547431 | 3555933 | 876525 |
| NMBU-41 | Fe-W-T | 7080248 | 2960661 | 4119587 | 892057 |
| NMBU-42 | Fe-W-T | 7252612 | 3408553 | 3844059 | 915918 |
| NMBU-43 | Al-D-T | 7210585 | 3494806 | 3715779 | 877790 |
| NMBU-44 | Al-D-T | 7274248 | 3509957 | 3764291 | 889104 |
| NMBU-45 | Al-W-C | 7549416 | 4066355 | 3483061 | 919495 |

**Table S19: Annotation of SNPs.**

**A B**

| **Type** | | |  | **Region** | | |
| --- | --- | --- | --- | --- | --- | --- |
| **Type (alphabetical order)** | **Count** | **Percent** |  | **Type (alphabetical order)** | **Count** | **Percent** |
| Downstream_gene_variant | 2466729 | 6,23% |  | Downstream | 2466729 | 6,24% |
| Initiator_codon_variant | 19 | 0,00% |  | Exon | 223900 | 0,57% |
| Intergenic_region | 23053964 | 58,24% |  | Intergenic | 23053964 | 58,27% |
| Intron_variant | 5550415 | 14,02% |  | Intron | 5535539 | 13,99% |
| Missense_variant | 99624 | 0,25% |  | Splice_site_acceptor | 348 | 0,00% |
| Non_canonical_start_codon | 4 | 0,00% |  | Splice_site_donor | 337 | 0,00% |
| Non_coding_transcript_variant | 5778833 | 14,60% |  | Splice_site_region | 16860 | 0,04% |
| Splice_acceptor_variant | 348 | 0,00% |  | Transcript | 5778833 | 14,61% |
| Splice_donor_variant | 467 | 0,00% |  | Upstream | 2486049 | 6,28% |
| Splice_region_variant | 19128 | 0,05% |  |  |  |  |
| Start_lost | 71 | 0,00% |  |  |  |  |
| Stop_gained | 1651 | 0,00% |  |  |  |  |
| Stop_lost | 1 | 0,00% |  |  |  |  |
| Synonymous_variant | 125203 | 0,32% |  |  |  |  |
| Upstream_gene_variant | 2486049 | 6,28% |  |  |  |  |

**Table S20: Mitochondrial genome annotation.**

| **Name** | **Start** | **Stop** | **Strand** |
| --- | --- | --- | --- |
| nad6 | 804 | 1331 | - |
| trnE(ttc) | 1332 | 1400 | - |
| cob | 1405 | 2547 | + |
| trnT(tgt) | 2548 | 2617 | + |
| trnP(tgg) | 2617 | 2682 | - |
| OH | 2852 | 3393 | + |
| trnF(gaa) | 3609 | 3677 | + |
| rrnS | 3678 | 4633 | + |
| trnV(tac) | 4633 | 4699 | + |
| rrnL | 4700 | 6268 | + |
| trnL2(taa) | 6270 | 6344 | + |
| nad1 | 6347 | 7303 | + |
| trnI(gat) | 7303 | 7371 | + |
| trnQ(ttg) | 7369 | 7440 | - |
| trnM(cat) | 7443 | 7511 | + |
| nad2 | 7512 | 8555 | + |
| trnW(tca) | 8554 | 8621 | + |
| trnA(tgc) | 8623 | 8691 | - |
| trnN(gtt) | 8693 | 8765 | - |
| OL | 8768 | 8798 | + |
| trnC(gca) | 8798 | 8864 | - |
| trnY(gta) | 8865 | 8933 | - |
| cox1 | 8935 | 10479 | + |
| trnS2(tga) | 10477 | 10545 | - |
| trnD(gtc) | 10553 | 10620 | + |
| cox2 | 10622 | 11305 | + |
| trnK(ttt) | 11309 | 11376 | + |
| atp8 | 11378 | 11578 | + |
| atp6 | 11539 | 12219 | + |
| cox3 | 12219 | 13003 | + |
| trnG(tcc) | 13003 | 13071 | + |
| nad3 | 13081 | 13428 | + |
| trnR(tcg) | 13419 | 13488 | + |
| nad4l | 13489 | 13785 | + |
| nad4 | 13812 | 15192 | + |
| trnH(gtg) | 15157 | 15225 | + |
| trnS1(gct) | 15226 | 15285 | + |
| trnL1(tag) | 15287 | 15356 | + |
| nad5 | 15348 | 820 | + |
